# Long-Term Survival of Hydrated Resting Eggs from *Brachionus plicatilis*

**DOI:** 10.1371/journal.pone.0029365

**Published:** 2012-01-09

**Authors:** Melody S. Clark, Nadav Y. Denekamp, Michael A. S. Thorne, Richard Reinhardt, Mario Drungowski, Marcus W. Albrecht, Sven Klages, Alfred Beck, Michael Kube, Esther Lubzens

**Affiliations:** 1 British Antarctic Survey, Natural Environment Research Council, High Cross, Cambridge, United Kingdom; 2 Israel Oceanographic and Limnological Research, Haifa, Israel; 3 Max Planck Institute for Molecular Genetics, Berlin-Dahlem, Germany; Macquarie University, Australia

## Abstract

**Background:**

Several organisms display dormancy and developmental arrest at embryonic stages. Long-term survival in the dormant form is usually associated with desiccation, orthodox plant seeds and *Artemia* cysts being well documented examples. Several aquatic invertebrates display dormancy during embryonic development and survive for tens or even hundreds of years in a hydrated form, raising the question of whether survival in the non-desiccated form of embryonic development depends on pathways similar to those occurring in desiccation tolerant forms.

**Methodology/Principal Findings:**

To address this question, Illumina short read sequencing was used to generate transcription profiles from the resting and amictic eggs of an aquatic invertebrate, the rotifer, *Brachionus plicatilis*. These two types of egg have very different life histories, with the dormant or diapausing resting eggs, the result of the sexual cycle and amictic eggs, the non-dormant products of the asexual cycle. Significant transcriptional differences were found between the two types of egg, with amictic eggs rich in genes involved in the morphological development into a juvenile rotifer. In contrast, representatives of classical “stress” proteins: a small heat shock protein, ferritin and Late Embryogenesis Abundant (LEA) proteins were identified in resting eggs. More importantly however, was the identification of transcripts for messenger ribonucleoprotein particles which stabilise RNA. These inhibit translation and provide a valuable source of useful RNAs which can be rapidly activated on the exit from dormancy. Apoptotic genes were also present. Although apoptosis is inconsistent with maintenance of prolonged dormancy, an altered apoptotic pathway has been proposed for *Artemia*, and this may be the case with the rotifer.

**Conclusions:**

These data represent the first transcriptional profiling of molecular processes associated with dormancy in a non-desiccated form and indicate important similarities in the molecular pathways activated in resting eggs compared with desiccated dormant forms, specifically plant seeds and *Artemia*.

## Introduction

Living organisms have evolved remarkable mechanisms for survival in unpredictable environments. Under extreme conditions that do not allow the maintenance of homeostasis, they may enter a state of reduced metabolism or developmental arrest, waiting for improved conditions that will allow the resumption of life activities, development and reproduction. Many organisms, from prokaryotes to mammals, have evolved the capacity to enter dormancy and exit from it. Surprisingly, common pathways have been identified, in spite of the diversity and complexity in the survival strategies in organisms displaying dormancy [1]–[3]. The term “dormant” or “dormancy” refers to a temporary suspension of visible metabolic activity or arrested development and encompasses the phenomena of diapause, quiescence or cryptobiosis [4]–[6]. Most often long-term dormancy is associated with desiccation [7].

Several organisms display dormancy and developmental arrest at embryonic developmental stages, including mammals [8]. Plants, rotifers, daphnids, copepods and *Artemia* are well known examples where dormancy is developmentally programmed in the form of seeds, resting eggs, ephippia or cysts [9]. In orthodox plant seeds, tolerance to desiccation is a programmed phase of embryological development which is regulated by abscisic acid and other hormones and initiated by maternal factors rather than environmental signals [10], [11]. During maturation, seeds undergo a series of declining water concentrations and acquire mechanisms for coping with osmotic adjustments (by accumulating carbohydrates), reactive oxidative species (by producing anti-oxidants) and structural changes by the production of small heat shock and Late Embryonenesis Abundant (LEA) proteins [1], [12]. Survival in a desiccated form is closely associated with the formation of an intracellular “glassy” matrix, with high intracellular viscosity restricting molecular mobility and life activities [1], [6]. Invertebrate desiccated cysts of *Artemia*, display some similar adaptations to those of plant seeds [13], [14]. Conversely, aquatic organisms, such as rotifers, cladocerans or copepods producing resting eggs or ephippia can survive for several decades or hundreds of years in a non-desiccated form [15]–[19], raising the question whether survival in the non-desiccated form of embryonic dormancy depends on pathways similar to those occurring in desiccation tolerant forms.

**Figure 1:**
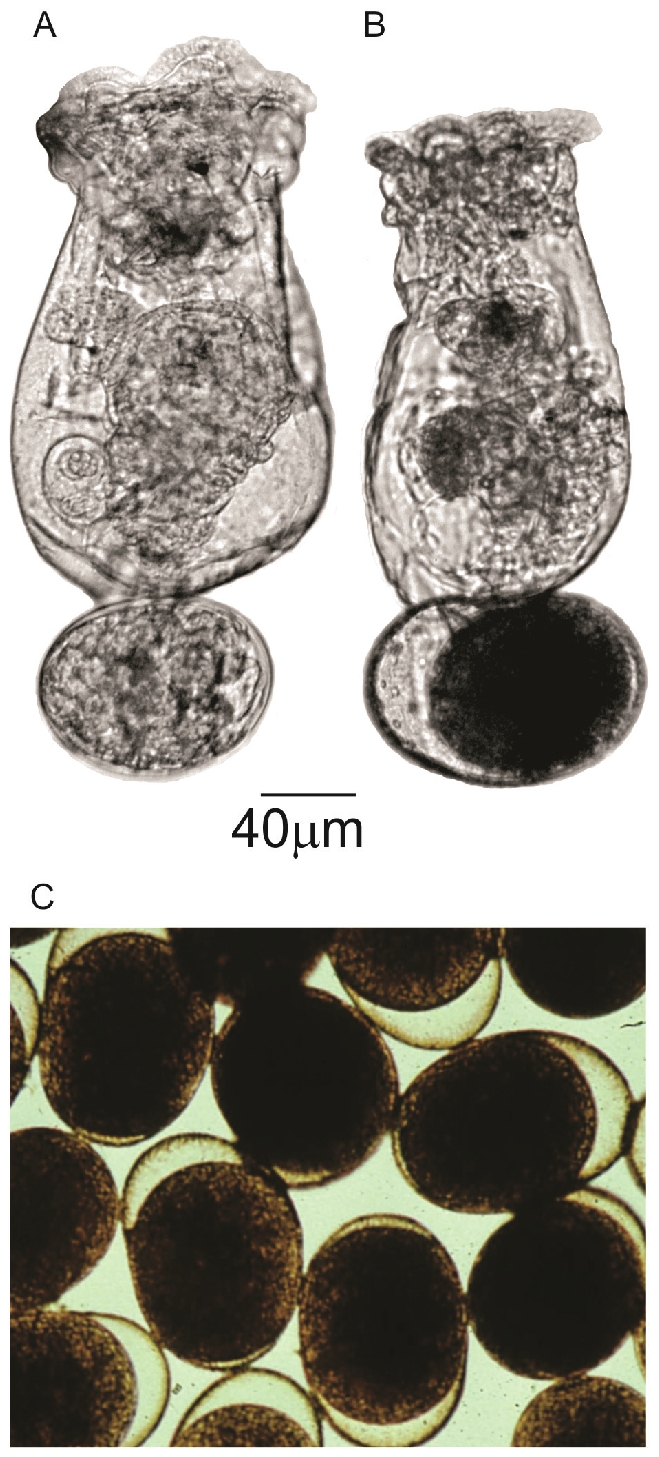
Comparison between amictic and resting eggs. Photographs of: **A:** An amictic female carrying an amictic egg with a developing embryo, **B:** A mictic female carrying a resting egg with an encased dormant embryo, and **C:** Resting eggs.

At least 15 monogonont rotifer species, including the rotifer *Brachionus plicatilis*, are known to enter dormancy or diapause through the formation of resting eggs by sexual reproduction [20]. The parthenogenetic (amictic) phase dominates the monogonont life cycle in the absence of males, but following certain environmental cues, sexual reproduction (the mictic phase) takes place via meiosis leading to the formation of resting eggs [9]. The dormant (or diapausing) resting egg is an encased embryo, differing morphologically from the non-dormant asexually (amictic) produced egg [20]–[22] (Figure 1) and in this form, can retain viability for decades [18]. Following specific cues, the resting eggs hatch after an obligatory dormant period and populate the water body through asexual reproduction [9]. The water content of resting eggs is around 70% [23] and in the natural environment they occur in the sediments of the littoral zone or saline ponds [16], [18]. However, rotifer resting eggs can also retain their viability after desiccation and lyophilisation, when their water content is reduced to ∼7% [23].

Genomic resources for developmentally arrested invertebrate embryos displaying dormancy are scarce with the exception of *Artemia* [24], [25] and the current study is the first for non-desiccated dormant embryos. In this study, gene expression profiles were generated for both *B. plicatilis* resting and amictic eggs using Illumina short read sequencing mapped against an existing EST transcriptome backbone [26]. This was a single time point replicated assay. Resting eggs first appeared to be carried by mictic females, 3–5 days after hatching and the resting eggs were collected 14–25 days after the hatching of the parental resting eggs. Amictic eggs were collected at the same time. The expression profiles were analysed to identify unique functions characteristic of each state. The genes identified in the resting egg transcription profiles were also compared to current data in other species, such as *Artemia* and plant seeds, to further understand potentially common processes involved in the maintenance of dormant forms.

## Results and Discussion

Illumina short read sequencing was used to generate transcription profiles of *B. plicatilis* resting and amictic eggs (details in Table S1). These two types of egg have very different life histories, with the resting eggs the result of the sexual cycle and are the dormant or diapausing form, whilst the amictic eggs are the parthenogenetic diploid products of the asexual cycle (Figure 1; also Figure 1 in [26]). The resting eggs can survive for decades in the dormant form [18], the original reference culture for this project dates back to 1981 (see Materials and Methods). Viability of the resting eggs used for RNA extractions analysed in this article were tested after 8 weeks of collection, having been stored in the dark at 4°C. Between 47–49% of the resting eggs hatched (Table S2). The proportion of viable eggs was probably higher, as previous results showed that resting eggs that do not hatch in the first attempt, hatch later under slightly different conditions [27], [28].

**Figure 2:**
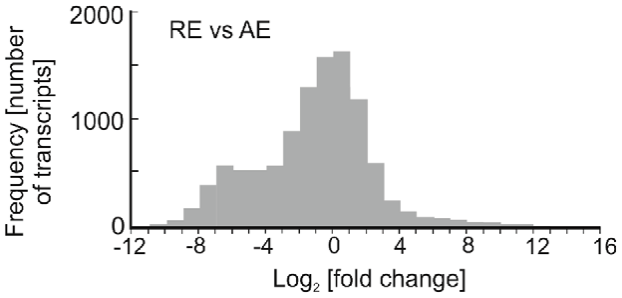
Two-way analysis comparing the number of transcripts (reads) and their respective fold change, between resting eggs (RE) and amictic eggs (AE). Each column shows the number of reads at the respective fold change. The reads in columns corresponding with range of −1 and +1 fold change, did not differ significantly between resting eggs and amictic eggs.

An analysis of the most commonly expressed transcripts in both forms identified striking similarities, but also some major differences (Table 1; Figure 2). There were commonalities represented by transcripts putatively involved in protein turnover, energy production in the form of genes involved in the mitochondrial respiratory chain and ATP synthesis and cytoskeletal elements. Hence there was a certain level of metabolic activity in both types of egg. Transcripts of the mitochondrial transport protein (voltage dependent anion channel or porin) were present in numerous copies in both types of egg, however, exact assignment of function is difficult as this is a multifunctional protein, crucial in many aspects of cell life and death. This protein forms a pore in the mitochondria and is the effective gatekeeper for entry and exit of metabolites, including ATP between this organelle and the cytosol. It also has important roles in protection against oxidative stress and the regulation of apoptosis [29]. Hence, this molecule could potentially be performing different functions in each of the egg forms.

**Table 1:** The ten most commonly expressed transcripts in the two egg forms.

Signature clone	Sequence match	Accession number	Expect	Function
***Resting eggs***				
sb103P0019I07_F.ab1	Ubiquitin carboxyl-terminal hydrolase	B4MJN2	0.11	Protein turnover
sbs02P0001I19_F.ab1	Voltage dependent anion-selective channel	E3TCS5	2.8E-12	Transport
rotifera-CL1Contig18	ATP synthase lipid binding protein	C1BPP0	1.1E-14	ATP synthesis
rotifera-CL5Contig1	HSP27	E5DVQ8	1.1E-35	Chaperone/stress protein
rotifera-CL90Contig1	Actin-related protein 2/3 complex	E2COG1	1.8E-05	Cytoskeleton
rotifera-CL485Contig2	CAMK/TSSK protein kinase	E1FMN8	1.7E-21	Cell differentiation
rotifera-CL1Contig28	Late Embryogenesis Abundant protein	D5IOZ6	9.4E-98	Desiccation tolerance
rotifera-CL2Contig3	Cytochrome C oxidase, subunit III	B1GYK1	8.9E-98	Electron transport
rotifera-CL57Contig1	Ferritin-like protein	C6JUM7	3.6E-25	Antioxidant
sbs01P0015E02R_.ab1	Peptidyl-prolyl cis-trans isomerise-like	Q9H2H8	7.6E-12	Accelerates protein folding
***Amictic eggs***				
sb103P0019I07_F.ab1	Ubiquitin carboxyl-terminal hydrolase	B4MJN2	0.11	Protein turnover
rotifera-CL2Contig5	Cytochrome C oxidase, subunit III	B1GYK1	4.9E-96	Electron transport
rotifera-CL485Contig2	cAMP-dependent protein catalytic sub-unit	E0VNF8	3.7E-21	Protein phosphorylation
rotifera-CL105Contig1	Tubulin ß chain	P11833	2.9E-216	Cytoskeleton
rotifera-CL2998Contig1	Ankyrin domain protein	C0QTZ9	2.0E-15	Transcriptional activator
rotifera-CL1Contig3	Selenophosphate synthetase	Q3HR35	9.0E-116	Cell cycle progression
rotifera-CL3Contig3	ATPsynthase F0 subunit 6	E2D7B0	1.6E-36	ATP synthesis
rotifera-CL52Contig1	Translation elongation factor 1α	C6L868	1.1E-231	Protein biosynthesis
rotifera-CL2956Contig1	NADH dehydrogenase subunit 3	B1GYK2	9.6E-12	Electron transport
sbs02P0001I19_F.ab1	Voltage dependent anion-selective channel	E3TCS5	2.8E-12	Transport

Representative clones have been annotated with putative functions. Different members of gene families (e.g. cytochrome c oxidase sub-units) have only been uniquely represented to provide a wider overview of the most common functions associated with each egg form. All expect values are less than 1.0e-12 except for that of ubiquitin carboxyl-terminal hydrolase (0.11). This was a very short, but accurate match, which is reflected in the high expect value. All assignments were manually verified.

An overview of the differences between the two types of embryo, can be obtained by examining those transcripts differentially expressed in each library via GO enrichment. These data revealed that resting egg transcripts showed cellular components and biological processes associated with protein turnover and degradation and molecular functions associated with, again, protein degradation, but also oxidoreductase activity (Table 2). The enriched functions linked to amictic eggs showed a very different composition indicating active cytoskeletal remodelling and cellular communication (Table 3). Molecular functions that predominated were associated with calcium ions, actin and cytoskeletal protein binding and kinase activity, whilst cell communication and signal transduction were the most significant biological processes.

**Table 2:** GO enrichment results of resting egg transcripts, showing most significant results for cellular component (denoted by C preceding the GO ID), biological process (B) and molecular function (M).

GO ID	Description	ratio	p-value	q-value
C:0000502	Proteasome complex	5.71	4.20E-21	8.08E-18
M:0004298	Threonine endopeptidase activity	5.92	2.32E-19	1.48E-16
C:0005839	Proteasome core complex	5.92	2.32E-19	1.48E-16
B:030163	Protein catabolic process	3.14	1.63E-16	7.85E-14
B:0006511	ubiquitin-dependent protein catabolic process	3.35	3.33E-15	8.01E-13
B:0043632	modification-dependent macromolecule catabolic process	3.35	3.33E-15	8.01E-13
B:0019941	modification-dependent protein catabolic process	3.35	3.33E-15	8.01E-13
B:0051603	Proteolysis involved in cellular protein catabolic process	3.35	3.33E-15	8.01E-13
C:005829	cytosol	2.88	5.67E-15	1.21E-12
B:0044257	Cellular protein catabolic process	3.26	1.19E-14	2.29E-12
B:0043285	Biopolymer catabolic process	2.57	4.25E-13	7.43E-11
C:0044445	Cytosolic part	3.36	9.81E-11	1.57E-08
B:0009057	Macromolecule catabolic process	2.18	1.58E-10	2.33E-08
B:0044265	Cellular macromolecule catabolic process	2.18	4.93E-09	6.76E-07
B:0009056	Catabolic process	1.58	7.97E-06	1.02E-03
C:0030529	Ribonucleoprotein complex	1.59	1.44E-05	1.73E-03
B:0044248	Cellular catabolic process	1.60	1.68E-05	1.90E-03
M:0016491	oxidoreductase_activity	1.40	2.13E-05	2.28E-03
C:0032991	Macromolecular complex	1.29	2.44E-05	2.46E-03
B:0043284	Biopolymer biosynthetic process	2.78	4.15E-05	3.98E-03

**Table 3:** GO enrichment results of amictic egg transcripts, showing most significant results for cellular component (denoted by C preceding the GO ID), biological process (B) and molecular function (M).

GO ID	Description	ratio	p-value	q-value
C:0005856	cytoskeleton	1.87	1.07E-17	2.69E-14
C:0016020	membrane	1.32	2.02E-16	2.54E-13
M:0008092	Cytoskeletal protein binding	2.34	8.39E-16	7.02E-13
M:0005509	Calcium ion binding	1.82	1.45E-14	9.12E-12
B:0007154	Cell communication	1.46	1.15E-12	5.78E-10
C:0044425	Membrane part	1.32	3.44E-12	1.44E-09
C:0042995	Cell projection	2.11	5.16E-12	1.85E-09
C:0044430	Cytoskeletal part	1.85	6.48E-12	2.04E-09
M:0003779	Actin binding	2.39	9.91E-12	2.77E-09
B:0007165	Signal transduction	1.43	3.18E-10	7.99E-08
C:0031224	Intrinsic to membrane	1.33	4.39E-10	1.00E-07
C:0005886	Plasma membrane	1.60	1.38E-09	2.88E-07
C:0016021	Integral to membrane	1.32	1.58E-09	3.05E-07
B:0051674	Localization of cell	2.31	1.04E-08	1.64E-06
B:0006928	Cell motility	2.31	1.04E-08	1.64E-06
C:0019861	flagellum	2.31	1.04E-08	1.64E-06
B:0032501	Multicellular organismal process	1.47	2.27E-08	3.35E-06
C:0015630	Microtubule cytoskeleton	1.76	3.25E-08	4.54E-06
M:0016301	Kinase activity	1.43	6.02E-08	7.95E-06
B:0007010	Cytoskeleton organization and biogenesis	1.71	2.78E-07	3.49E-05

In-depth analysis aided by manual verification revealed greater detail on the cellular activities of resting and amictic eggs. Full details of these transcripts with associated BLAST sequence similarity data and putatively assigned functions are given in Tables S3 and S4, with an extraction of the function data summarised in Table 4. These are discussed in more detail below.

**Table 4:** List of putative functions assigned to transcripts differentially up-regulated in resting and amictic eggs (extracted from data in Tables S3 and S4).

Putative function	Resting eggs	Amictic eggs
Antioxidant	29	
Chaperone systems	12	
Transcription factor	8	
Oxidoreductase	7	
Nucleotide biosynthesis	7	
Late Embryogenesis Abundant protein	4	
Apoptosis	4	
Translation	4	
Chromosome structure, replication and transcriptional regulation	3	
Trehalose biochemical pathway	3	
Cell adhesion/neuronal function	2	
Protein damage repair	2	
Amino acid metabolism	2	
DNA replication	2	
Mitochondrial distribution	2	
RNA metabolism	2	
Cell proliferation	2	
Protein modification	1	
Accelerate protein folding	1	
Molecular facilitator	1	
**Metabolism**	**12**	**2**
**Transporter**	**8**	**1**
**Protein turnover**	**8**	**1**
**Signalling molecule**	**8**	**8**
**Immune function**	**6**	**3**
**Lipid metabolism**	**4**	**7**
**Energy production**	**3**	**2**
**Transcription**	**2**	**1**
**Cell cycle progression/development**	**2**	**5**
**Cell differentiation**	**1**	**3**
Neurosecretion		1
Cell adhesion		2
Cilia component		3
Development		5
Proteolytic enzyme		8
Cytoskeleton/muscle		26

Annotation of function was carried by manual verification. The functions are notionally listed in order of abundance. Shared functional assignments are highlighted in bold. For a full listing and description of differentially expressed transcripts, see Tables S3 and S4.

### Transcripts up-regulated in resting eggs

Putatively identified transcripts in this list (Table 2) share many features in common with previous studies on dormancy and diapause of desiccated forms such as *Artemia* cysts and plant seeds [12], [13], in particular the preponderance of antioxidants, oxidoreductases, chaperones and late embryogenesis abundant proteins (LEAs).

#### Antioxidants

These are represented by database matches to ferritin, peroxiredoxin, catalase, glutathione-s-transferase and the superoxide dismutases. Although these genes have been shown to have other functions, such as increased lifespan [30] and iron sequestration [31], a major role of these enzymes is their action against reactive oxygen species (ROS). DJ-1, in particular has numerous functions including transcriptional regulation, protease activity and mitochondrial regulation, but given the biological context, allied to the catalogue of other up-regulated genes, the most probable function is protection against oxidative stress [32]. ROS are toxic in all life stages as they directly damage DNA, lipids and proteins, but are especially problematic for dormant forms. In plant seeds desiccation causes loss of control mechanisms that maintain low ROS concentrations and therefore antioxidant activity has great importance [33]. However, recently it has been shown that ROS play an important role in cell signalling particularly via post-translational modification of cysteine residues. Biological processes affected include chemotaxis, cell proliferation and neurogenesis [34] and it may well be that a certain level of ROS activity is beneficial to the resting eggs.

Oxidative stress leads to the accumulation of toxic compounds such as aldehydes that serve as intermediates in metabolism but may also be toxic to cells and therefore their amount must be tightly regulated. The higher expression of aldo-keto reductases may facilitate increased oxidative and heat stress tolerance [35], and protection of mitochondria against oxidative stress is suggested by higher levels of retinol dehydrogenase 13 (Table S3) [36].

#### Chaperones

Many proteins of the cellular chaperone systems are expressed ubiquitously in the normal cell state to aid in the folding of native polypeptides and their translocation to different cellular compartments [37], [38]. However, during the stress response, they may be up-regulated to further assist mis-folded proteins to attain or regain their native states and also target degraded proteins and regulate their removal from the cell, thus preventing the formation of cytotoxic aggregates [39]. The fact that elements of this system are up-regulated in resting eggs, supports the supposition that this state imposes a “stress” on rotifer cellular homeostasis. This is exemplified by the up-regulation of the HSP70 pathway (HSP70 with DnaJ co-chaperones and the HSC70/HSP90 organising protein), which has long been associated with the cellular stress response [37]. In *Artemia* cysts, the small heat shock protein p26 is present in high abundance and thought to play a major role in combating stressful conditions. This is a multifunctional molecule which possesses molecular chaperone activity, inhibits cell division by disruption of the mitotic apparatus, influences transcription levels and inhibits apoptosis (reviewed in [13]). All functions of which are essential for survival in the dormant state. The highest sequence similarity results for the small rotifer heat shock proteins identified here were to *Artemia* HSP21, but sequence homologies between small HSPs are generally low. To date, no orthologue of artemin, the ferritin superfamily member found exclusively associated with diapause and desiccation in *Artemia* [40], has been identified in any other species including the rotifer. This is a multifunctional protein that also acts as a chaperone depending on cellular requirements [13]. Small heat shock proteins may play a particularly important role in dormant organisms, as they act as ATP-independent chaperones, thus preventing protein aggregation in stressful conditions without the expenditure of important ATP reserves [41].

The specific expression of genes encoding small heat shock proteins and HSP70 have also been reported in association with dormancy in plant seeds [2], [42] and diapause in insects [13].

#### Oxidoreductases

This category comprises a range of different enzyme families including the cytochrome P450s, alcohol dehydrogenases and selenium binding proteins. These proteins play critical roles in the intermediate metabolism of many cellular substrates such as lipids, amino acids and carbohydrates, but are also major players in detoxification processes and degradation of xenobiotics, in the latter case, this is particularly true of the cytochrome P450 family. They are involved in the regulation of hormones, act as signalling molecules and sense the redox status in metabolic or transcriptional processes. Therefore they regulate vital cellular processes [43], [44]. Which role predominates in resting eggs cannot be determined purely from transcriptional profiles and certainly for such complex multifunctional families, the answer will almost certainly be a combination of functions. The CYP450 family is associated with synthesis of abcisic acid (P450 707) promoting dormancy in plant seeds [11] and dauer formation in *C. elegans* [45]. It is also up-regulated during diapause in the insect, *Allonemobius socius* [46]. Cytochrome p450s accumulate in *Artemia* cysts [47] indicating once again that potentially similar mechanisms may be operating in both the rotifer and *Artemia* dormant states.

#### Late embryogenesis abundant proteins (LEAs) and trehalose

These proteins were originally identified in plant seeds during the late stages of embryonic development [48] and are associated with desiccation tolerance throughout the life cycle of all major plant taxa [12]. They have subsequently been described in a number of naturally anhydrobiotic organisms and differentiated into seven groups, the most numerous of which are the Group 3 LEAs, which are the main ones found in non-plant species (for review see [49]). They are a family of highly hydrophilic proteins which are thought to act as a “molecular shield” playing a role in anti-aggregation and protein stabilisation [49]. Two Group 3 LEAs have previously been characterized in *B. plicatilis* and Q-PCR analyses showed their expression was significantly up-regulated in resting eggs and females with resting eggs [26], [50]. The presence of these LEAs in the transcription profile of resting eggs clearly substantiates this previous result and confirms their importance in both the maintenance of this physiologically “inert” state and desiccation tolerance, if this occurs. Although in general rotifer resting eggs are not desiccated, given particularly adverse conditions they can survive in a desiccated state. This could potentially explain the presence of the LEA proteins and also DNA repair enzymes as a preparative measure.

The LEAs are the only desiccation tolerance proteins identified in *B. plicatilis* to date. Trehalose is the classic anhydroprotectant (first identified in *Artemia* cysts [51], [52]), which stabilises biomacromolecules and the membrane bi-layer structure under stressful conditions [53]. However the transcription profiles only indicate the presence of trehalase in resting eggs. This is the enzyme which breaks down trehalose. To date, only very small amounts of trehalose have been measured in *B. plicatilis* resting eggs [54] with no up-regulation of trehalose production [26]. This is further verified by our data. The presence of trehalase could indicate that the small amount of trehalose present in resting eggs is actually being broken down to glucose. This could then be used as a primary energy source, prior to lipid utilisation from the lipid bodies described below. Non-reducing sugars such as trehalose, sucrose and oligosaccharides, in relatively high concentrations, are characteristic of orthodox plant seeds and provide an increased tolerance to abiotic stress [2].

#### Additional putative functions

Whilst resting eggs may be regarded in physiological terms as relatively inert or dormant, the expression profile data indicate otherwise. There are putative gene identifications in many categories, including those associated with transcription factors, cell division and cell proliferation, production of new RNAs and proteins via transcription, translation and RNA metabolism, protein turnover and general cellular metabolism. These cannot all be discussed in detail, but certain transcripts warrant highlighting as potential candidates for further investigation, either in their own right or because of the indication of biochemical pathway activation in the resting egg.

#### Apoptosis and cellular differentiation

Stressful conditions often lead to programmed cell death. There are three transcripts present; BAP-31, an integral ER membrane protein, misato homolog 1 and peptidase C14, a caspase catalytic subunit. All are key players in apoptosis, however processes concerned with cell death are not conducive to long-term survival and recovery during dormancy. In *Artemia*, whilst a similar complement of genes is present in the apoptotic pathway, these are not subject to the same control mechanisms as mammalian systems [55]. Potentially this is the case here, especially as it has been found that caspases in other invertebrates (e.g. *C. elegans* and *Drosophila*) indicate that they may be involved in non-lethal roles such as cellular differentiation processes [56]; cell proliferation, adhesion and differentiation [57]. Indeed there are other transcripts present which are involved in cellular differentiation and proliferation processes in invertebrates. These include headcase which is involved in adult morphogenesis in *Drosophila* [58], ependymin, a cell adhesion molecule up-regulated during regeneration in the echinoderm *Holothuria glaberrima* [59]; inorganic pyrophosphatase associated with larval development in the round worm *Ascaris lumbricoides* [60] and tetraspanin, which has been termed a molecular facilitator [61] and is expressed in diverse developmental stages in the tobacco hornmoth *Manduca sexta* [62]. There are also transcripts present which have been implicated in neuronal functions, such as the serine/threonine protein phosphatase which shows highest sequence similarity to PPEF-1 in *Homo sapiens* and sulfotransferase [63]. The presence of these transcripts is particularly interesting as they imply continued morphological development in the egg.

#### Lipid metabolism

Also of significant interest for future research are genes potentially involved in lipid metabolism. Methyltransferase-like 7 transcripts are involved in lipid droplet formation [64]. Dormancy has been associated with a function for lipids and lipid metabolism and lipid droplets were reported to occur in rotifer resting eggs [22]. Lipid metabolic pathways are up-regulated in the dormant dauer stage of *C. elegans* [65] and lipids serve as the main energy source during hibernation [66]. Unexpectedly Pauwels et al [67] did not find differences in the triglycerol content between subcutaneous and dormant eggs in *Daphnia magna*, however, newborn *Brachionus calyciflorus* females hatching from resting eggs had many more lipid droplets in their tissues than similar sized newborn females from parthenogenetic eggs [68]. Cathepsin L- protease was found essential for yolk processing and degradation during embryogenesis in *C. elegans* [69] and possibly may also have a function in eggs, as several transcripts for cathepsin-L-like cysteine proteinase were found to be up-regulated in resting eggs but also in amictic eggs.

To add to this novel identification is the presence of other genes which potentially protect lipid stores. The Krüppel-like transcription factor; Copeb-prov protein is up-regulated during lipid peroxidation in rats [70] and certain glutathione-s-transferases in *Drosophila* are known to be involved in combating oxidative stress and metabolism of endogenously formed lipid peroxidation products [71]. Transcripts for a selenium binding protein were up-regulated in resting eggs. Selenoproteins function as antioxidants and can decrease lipid peroxidation in *Drosophila* [72] and a selenoprotein was found to be up-regulated during diapause in female *Culex pipiens* [73].

#### Defence and protection

Hydrated resting eggs have specific external protective layers that are not found in amicitic eggs (Figure 1B) [22]. They are exposed during their long dormant period to bacteria and fungi that may affect their survival and reduce hatching [74]. A few transcripts associated with the immune function were up-regulated in resting eggs. These include Toll-like receptor 3 that could serve against viral infection [75], peptidase C14, a caspase mediating programmed death [76], F-box and WD-40, associated with the cellular proteolytic machinery and dauer development [46] and lanthionine synthetase C-like protein, representing a family associated peptide modifying enzyme components in eukaryotic cells and immune response to protozoan infection in an oyster [77]. F-box/WD-40 was also expressed in *Artemia* diapause destined embryos [47]. The higher expression of genes related to defence and protection was demonstrated for dormant plant seeds [42].

Finally, of particular interest in this list of differentially expressed genes was the presence of transcripts involved in the production of messenger ribonucleoprotein particles. These were reported in both *Artemia* and plant seeds and are associated with stored mRNA pools. These stabilise RNA and inhibit translation, providing a viable source of useful RNAs which can be immediately activated on the exit from dormancy [78], [79].

### Transcripts up-regulated in amictic eggs

Although there were some shared functional categories between the transcripts differentially expressed in resting and amictic eggs, such as metabolism, signalling, immune function etc, the relative balance of these in each type of egg differs and there was a very distinct partitioning of key genes and functions (Table 4, Table S4).

#### Cytoskeleton

By far the vast majority of identifications were transcripts potentially involved in the cytoskeleton and development and functioning of muscle. These included smoothelin, a muscle cell specific cytoskeletal protein, which is developmentally regulated in vertebrates [80]. The phenotype of smooth muscle cells varies depending on origin, location or function and can change during maturation. Smooth muscle cells express a combination of proteins characteristic of the degree of maturation and smoothelin is one of these markers. Transcripts were also present for calponin, a component of smooth muscle and numerous myosins. The latter are an extensive gene family, the functions of which are largely unknown but have been implicated in large repertoire of cellular functions including cell migration, cell adhesion, organelle transport, receptor mediated and fluid phase endocytosis, mRNA transport and transcription, along with their interactions with the cytoskeleton (e.g. [81]. Increased activity of the cytoskeleton has direct links to and indeed, a requirement for, cell signalling. In the list of differentially expressed genes in amictic eggs (Table S4), calmodulin, in particular, mediates the control of a large number of enzymes and other proteins by calcium. It directly interacts with another transcript (enkurin) present in this dataset. Likewise, the CAPSL protein transcript contains an EF hand domain, which is characteristic of signalling molecules including calmodulin.

#### Morphological development

The preponderance of cytoskeletal genes in the transcription profile of amictic eggs accords with the morphological development of the egg into a juvenile rotifer. This is substantiated by the detection of transcripts specifically involved cell proliferation and adhesion such as ependymin and countin, which regulates cell adhesion and controls cell numbers [82]. There were also transcripts active in development, the prime example being notch. This gene family is highly conserved throughout the animal kingdom and is involved in diverse developmental and physiological pathway lineage decisions, boundary segregations, neurogenesis and cell fates [83]. There were signals of increasing neuronal development with the identification of innexins. These are involved in neurogenesis and are involved in neuronal development in the leech [84]. Of particular note was the presence of the prohormone vasotocin. Relatively little is known about the functioning of neurosecretory cell types in invertebrates. Database mining shows that annelids and molluscs have preserved a large fraction of vertebrate-type neuropeptides, including vasotocin. However, this hormone is not ubiquitous to invertebrates, being absent in both *Drosophila* or *C. elegans*. This prohormone is expressed in neurosecretory cells and involved in the neuronal development of annelid worms [85]. The identification of this transcript in the rotifer is clearly of interest in terms of organism development, but will also be of more general interest to evolutionary biologists. Complementary to the work on *Artemia*, was the detection of a transcript with high sequence similarity to SPARC (secreted protein, acidic, rich in cysteine): also termed osteonectin and BM-40. This is a calcium binding glycoprotein expressed in extracellular matrices of various cell types undergoing morphogenesis, development, remodelling and wound healing. This gene was identified in *Artemia* and whilst faintly expressed in embryos, was considerably up-regulated in prenauplii and nauplii [86]. Whilst a developmental role is proposed for this protein in *Artemia*, phylogenetic analysis showed distinct separation of this sequence into vertebrates and invertebrates, suggesting that there are, as yet, novel unidentified functions in invertebrates.

#### Lipid metabolism

Finally, was the identification of a group of transcripts potentially involved in lipid metabolism. While such genes were identified in resting eggs (which have already been described), the amictic eggs contained a different sub-set. Calveolin is one of a limited group of proteins associated with lipid bodies [87]. Endogenous calveolin moves to lipid bodies in response to the accumulation of lipids and hence its presence substantiated findings of lipid body accumulation in rotifers [68]. However, the cellular location of this protein is not static; in regenerating liver calveolins move from the plasma membrane to newly formed lipid bodies suggesting a role for calveolin in lipid transport to/from lipid bodies and general intra cellular homeostasis of lipids [87]. Additionally, in the differentially expressed genes was the presence of an ecdysteroid-regulated 16 kDa protein. This molecule contains a lipid recognition domain and has been shown to be involved in protein uptake in larval fat body in *Drosophila* [88]. Also present was a delta 6 fatty acid desaturase. This is a component of the lipid metabolic pathway that catalyzes biosynthesis of highly unsaturated fatty acids from precursor essential polyunsaturated fatty acids such as linoleic acid. These support previously published results on synthesis of unsaturated acids in rotifers [89]. Highly unsaturated fatty acids play pivotal roles in many biological functions. Combined with the results of lipid metabolism genes in resting eggs, these identifications indicated the potential dynamic nature of lipid metabolism in rotifer life history stages, providing candidate genes for future analyses of the requirements for entry and exit into dormancy of this species.

#### Comparative analyses with other species

Considering the results, the most surprising, was the identification of numerous transcripts in the resting eggs that encode genes for essential biochemical pathways (Table 4). This is counter-intuitive, given the knowledge that *B. plicatilis* resting eggs can remain dormant for tens of years and still remain viable [16], [18], [19]. However if this biochemical turnover were in a continual state then energy stores would rapidly become depleted and the eggs inviable, a situation clearly at odds with long term survival in the dormant state. While there are no reports on metabolic rates of rotifer dormant eggs, the decrease in embryo size with time in sediments supports the notion that dormant or diapausing eggs are metabolically active albeit at a very low level [19]. Also observations showing survival in resting eggs collected from pond sediments, indicates a dependency of long-term survival on environmental conditions [19]. However there are a small number of examples of hydrated resting eggs from marine and freshwater systems which exhibit long term survival in this dormant form [90]. In particular is the example of *Artemia*, where intensive investigations of the metabolic activity of hydrated eggs under anoxic conditions failed to reveal any measureable metabolism over four years [91]. The mechanism by which these species can survive and remain viable under such conditions for long periods of time is, as yet, unknown.

It is also curious that there was an increased complexity of functions associated with resting eggs compared to amictic eggs. One reason to explain why there is transcriptional activity associated with the resting eggs is that when they are formed, they are not “mature” enough to survive a dormant period. This is substantiated by findings that during this maturation period the eggs cannot be hatched and will not survive the dormant period, similar to plant seeds [27], [42]. It is suggested that during this obligatory diapause stage, metabolic changes can occur, but to date there is no description of their nature. These transcription profiles are potentially the first snapshot of this period.

### *Artemia*

In response to adverse conditions, embryos of the *Artemia* enter an anhydrobiotic dormant state in the form of a cyst. This is associated with developmental arrest and cessation of virtually all metabolism and DNA, RNA and protein synthesis [92]. The organism may remain in this ametabolic state for decades, but still remain viable if the appropriate environmental signals are applied [93]. On rehydration, the cysts almost instantaneously resume development and hatch as naupilus larvae. Dormancy in this species has been studied at various levels for almost 30 years and is one of the best studied systems in this regard. In particular work has concentrated in two inter-linked areas: metabolic arrest and lack of activation of programmed cell death (apoptosis).

Transcription and translation studies showed that dormant embryos contain significant amounts of mRNA. Comparisons of active and dormant organisms indicated that both states contained similar quantities of translatable mRNA, hence there was no net degradation of mRNA pools during dormancy. The conclusion being that control was at the translation level with an arrest of protein synthesis [94], [95]. The exact nature of how this is effected in the embryo (in terms of cell signalling) has yet to be accurately defined, but there are almost certainly several factors involved.

Early measurements on nucleotide metabolism showed an 80% reduction in ATP levels in first hour of embryonic anoxia [96]. This dramatic reduction in cellular energy levels could potentially explain the rapid *in vivo* arrest, without the detail of the mechanisms. Dormant embryos contain large quantities of messenger ribonucleoprotein particles [78] and it is thought that their association with the stored mRNA pool performs two roles: mRNA stabilisation and inhibition of translation [79]. This is potentially allied to the finding of an extension of mRNA half-lives and transcription initiation reduced to approximately 90% of normoxic levels in short-term treated anoxic embryos [97]. p26, a small heat shock protein is also mobilised from the cytoplasm to the nucleus during anoxia and reversed under normoxia [98]. This molecule possesses molecular chaperone activity, but has also been implicated in transcriptional arrest [99]. No doubt, it is a combination of factors that lead to the successful induction of dormancy, but with regard to the maintenance of this state, programmed cell death must also be inhibited.

The latter is an automatic reaction of mammalian cells to an environmental challenge. This produces an uncoupling of respiration and release of cytochrome c in the mitochondria, with a sudden increase in permeability of the inner mitochondrial membrane to solutes with molecular masses of up to 1500 Da. This is known as the mammalian mitochondrial permeability transition. Cytochrome c in mitochondria is essential for oxidative phosphorylation, but its release into cytoplasm initiates assembly of the apoptosome, the molecular machinery activating the caspase 9 pathway and programmed cell death. Mitochondrial permeability and caspase activation in *Artemia* are not subject to the same control mechanisms as mammalian systems [55]. *Artemia* caspase activation is not dependant on cytochrome c, but is regulated (at least partly) by nucleotide concentrations. Therefore similarly to metabolic arrest, lack of caspase activation is consistent with energetic trade-offs occurring as a result of rapid and dramatic energy limitation [55]. Putative transcripts for BAP-31 and a caspase were identified in the resting eggs and these are obvious candidates for future studies to further our understanding of cell death processes and their control in different species.

### Plant seeds

The relationship of the resting eggs to plant seeds, although sharing similarities with *Artemia*, is proving slightly more complex to dissect. Like *Artemia*, dry mature seeds contain a large number of stored mRNAs and again translation is thought to be the major level of control [100]. These transcriptionally competent mRNAs are not thought to be actively transcribed *in vivo* due to severely reduced moisture levels [101]. Whilst over half the stored mRNA species are remnants from embryogenesis and seed maturation, the others provide important RNA templates for protein synthesis during the early stages of germination. Indeed the most highly expressed 2–3% of mRNAs in stored seeds encode functions associated with metabolism, protein synthesis and degradation [101]. Hence when conditions are suitable for germination, the cellular machinery can rapidly produce the required proteins with minimal investment in transcription. However, several recent studies have detected low levels of both transcription and translation in plant seeds (reviewed in [102]). Seeds can exist in many states of partial imbibition, therefore “dry”, in at least some plant species, may mean low-hydrated with regional compartmentalisation of hydration states. This could mean that seeds maintain a number of more hydrated areas with limited capacity for complex cellular interactions. How relevant this is to the final germination process is still under evaluation, but offers tangible opportunities for laser micro-dissection studies [102].

The work described here and many of the reference studies involve analyses of mRNA transcription profiles. These are undertaken with the *a priori* hypothesis that such profiles and changes in levels of transcript expression are indicative of the general functioning and responses of the organism under study. How changes in gene transcript abundances translate into production of proteins and whole animal functions is an area of considerable interest and clearly impact on the relevance of our conclusions. A recent investigation in a prokaryote (*Mycoplasma pneumonia*) indicated that regulation of gene expression was largely decoupled from protein dynamics, but this did depend on which genes were analysed, with strong correlations recorded for some, e.g. heat shock proteins [103]. The situation in eukaryotes may differ. Earlier work on whole animals showed a general correlation of mRNA synthesis with protein production, but the timing and magnitude of protein production was not consistently predicted by mRNA concentration [104]. A more extensive tissue-specific study noted that differences in gene expression reflected well-established tissue-specific metabolic requirements, suggesting that measures of gene expression accurately reflected changes in proteins and their phenotypic effects [105]. More recent data have shown that expression levels between orthologous proteins and mRNAs, were generally positively correlated [106], in fact, much better than previously thought with around 40% of the variance in protein abundance explained by mRNA levels [107]. This new understanding has been possible due to methodological improvements and the ability to survey increasingly large numbers of transcripts/proteins in the same experiment [107]. Almost certainly, our abilities to undertake such fine-scale studies will increase in the future, enabling us to disentangle the complex relationship between mRNAs, proteins and whole animal functioning.

### Summary

Amictic eggs showed a consolidated transcriptional profile associated with cell proliferation, cytoskeletal remodelling and development, which matches the physiological changes that the egg undergoes as it matures into a juvenile rotifer. However resting eggs revealed a more a complex transcriptional profile associated with many different cellular processes. Knowledge of rotifer biology and comparisons with other species indicate several possible reasons for this observation. The first of which is that the resting eggs are still maturing. Secondly there is abundant evidence from both *Artemia* and plant seeds that there is a preparative storing of useful transcripts with a cessation of protein synthesis, so that on emergence from dormancy the organism only has to activate translation for the egg to develop, not both processes of transcription and translation (which is far more energetically costly, when nutrients are limiting). It is particularly interesting to note that the transcription factors identified were all present in the resting egg state and are often associated with cell proliferation and differentiation in other species (e.g. MYND and SET domains, and Krüppel-like factors [108], [109].

Resting eggs represent forms entering dormancy during embryonic development but are not necessarily desiccated. The main characteristic of this form of dormancy is the suspension of development. In forms undergoing desiccation such as *Artemia* cysts or orthodox plant seeds, metabolic activity will be dramatically reduced due to the intracellular glassy state and high viscosity. It is not clear how metabolism is suspended in hydrated forms, such as rotifer, cladoceran and crustacean resting eggs but its duration may depend on energy stores such as lipids, protection against invasive microorganisms and possibly an altered programmed cell death pathway. There are clearly critical adaptations to survival similar to other species undergoing dormancy or diapause, producing abundant amounts of chaperones, antioxidants, and LEA proteins but more studies are needed to unveil the regulation of these processes in dormant embryos of invertebrates. Indeed, although this rotifer study does represent a substantial increase in expression data related to the dormancy process, it is a single time point assay and to understand what may be going on more detailed sampling may be required in the future. The transcriptional basis of dormancy seems to differ substantially between species, reflecting the multiple evolutionary origins and life strategies. Yet, dormancy always involves reduced metabolism, up-regulation of stress resistance and upholding of cellular or protein structure. Investigations into long term survival in hydrated embryos could lead to developing methods for long term preservation of hydrated cells, in analogy with achievements in the dry preservation of human cells, which stemmed from investigations into long-term survival and desiccation tolerance of *Artemia* cysts [110].

## Materials and Methods

### Rotifer samples

*Brachionus plicatilis* rotifers were hatched from resting eggs produced in the laboratory from rotifers collected at a seaside pond in Atlit, (40 km south of Haifa, Israel) in 1981. Some of the resting eggs were hatched in 2003 and resting eggs produced from them were stored in the laboratory at 4°C and hatched in 2005. Resting eggs that were hatched in 2005 served to produce several batches of resting eggs that were stored at 4°C. Four different cultures were started, originating from four different batches of resting eggs. These eggs were hatched in an illuminated culture room (at 25°C) and the salinity of the seawater culture medium was 10 ppt. In general, rotifers were cultured as previously described in [26], [111]. Resting eggs first appeared to be carried by mictic females, 3–5 days after hatching and the resting eggs were collected 14–25 days after hatching of the parental resting eggs. In order to assess the viability of the resting eggs that were collected for RNA extraction, resting eggs from two replicate cultures, were hatched after 0, 2, 4, 6 and 8 weeks of collection. Between 47–49% of the resting eggs hatched after 8 weeks of storage in the dark at 4°C (Table S2). The proportion of viable eggs is probably higher, as previous results showed that resting eggs that do not hatch in the first attempt hatch later, under slightly different conditions [27].

### Collection of resting eggs

Resting eggs were collected from the bottom layer of the culture flasks. Aliquots of 10 ml collected from bottom layer were placed in a large petri-dish and resting eggs were picked manually and removed from the amictic eggs and rotifers found in this layer. RNA was extracted immediately. Four biological replicate experiments were performed, with each replicate originating from resting eggs produced by a different culture. About 5,000 resting eggs were used for RNA extraction from each biological replicate sample.

### Collection of amictic eggs

Samples of amictic females (FA) were manually picked from each culture, when the resting-egg production rate was at its peak. An amictic female was recognized by the relatively large opaque eggs it carried and only females with eggs were manually collected. Rotifers for each replicate were suspended in fresh sterile seawater (10 ppt) and sieved with 60 µm plankton nets, washed with sterile diluted sea-water medium (10 ppt) and re-suspended in 10–15 ml of sterile seawater (10 ppt) in a 15 ml round bottom disposable vial. The amictic eggs (AE) were collected by intensive up and down movement with a five ml pipette tip (‘re-pipetting”), of five ml samples containing amictic females. In this way, the amictic eggs were shed from the females and could be collected from the bottom of the vial. They were manually cleaned from newborn females that continuously hatched from the eggs. Hatching of the amictic eggs was stopped by adding ethanol (10% final concentration), whereby the eggs settled at the bottom of a vial, the supernatant was removed, and the sample was frozen in liquid nitrogen. The eggs were stored at −70°C until RNA extraction. Four biological replicate experiments were performed with approximately 5,000 eggs used for RNA extraction from each biological replicate sample.

### Library construction and sequencing

RNA was extracted from resting and amictic eggs using TRIzol reagent (Invitrogen) according to manufacturer's instructions. Total RNA was extracted from each one of the four biological replicates of amictic eggs and four biological replicates of resting eggs. A pooled RNA sample was formed from equal amounts of RNA from each replicate of resting eggs and similarly, a pooled RNA sample was formed from equal amounts of RNA from each replicate of amictic eggs. After measurement and quality control 1.5 µg of total RNA (from the pooled RNA sample of resting egg sample or pooled RNA sample of amictic egg RNA) was used as template for cDNA synthesis, by the Mint cDNA synthesis kit (Evrogen, Russia) following the manufacturer instructions. The amplified cDNA was purified (QIAquick PCR Purification Kit, Qiagen). The cDNA was measured and quality checked by gel electrophoreses. An amount of at least 8 µg of cDNA per replicate with a size distribution between 0.5–3 kbp was obtained. Library generation and sequencing were performed using the Illumina sequencing platform (Genome Analyzer, Illumina) according to the manufacturer's instructions for shotgun sequencing. Each of the two sequenced libraries represented approximately 15,000 individuals for each of the amictic eggs and resting eggs. A full description of the sequencing protocols is described in [111]. We previously reported on the construction and Illumina sequencing of 13 libraries from different life stages of *Brachionus plicatilis* and the current study focuses on two of these libraries [111].

### Analyses of data from Illumina sequencing

Sequences were extracted using Gerald from the Illumina Pipeline v1.0 with all possible basecalls and with standard QC turned on (chastity filter > = 0.6). The sequences (reads) from the libraries were aligned against rotifer ∼18,000 EST sequences [26] using SOAP [112] allowing 2 mismatches within 32 bp. Prior to that, reads containing more than two Ns were filtered and discarded. For each library, the sum of all aligned reads of their specific contig was calculated (equal to transcripts in this case), not keeping track of the uniqueness. Because the number of total reads varied between libraries (due to technical differences stemming from PCR amplification, library preparation etc.), a simple scaling method was used to facilitate comparisons between them (described in [111]). The analysis for differential expression was carried out using R [113] with standard chi-square test, Fisher exact test and a self implemented version of Audic and Claverie bayesian approach [114]. A transcript was considered to be changed differentially if it matched the following cases:

- state1/state2< = 0.5 OR state1/state2> = 2.0
- #{reads.state1}> = 50 OR #{reads.state2}> = 50
- one of the three statistical test p-values< = 0.01

The backbone contigs (and therefore short read mappings) were annotated via database searches using Blastx [115] against the NCBI database with matches annotated for all scores with an expect score in excess of 1e-10. All transcript matches presented in the results were manually verified. Sequences with a database match were then further annotated using GO [116]. Further sequence manipulation was carried out using the EMBOSS suite of programmes [117]. In order to classify the genes by functional categories of GO terms, enrichment analysis was performed using the hypergeometric distribution function. Multiple test corrections of p-values were performed by q value calculations using the R-package “qvalue”. Significant-thresholds for selections were p-value< = 0.05, q-value< = 0.1. Verification of the two transcription profiles were performed across the biological replicates using Q-PCR, evaluated and described for 13 libraries [111] and is shown here for the libraries formed from amictic eggs and resting eggs (Figure S1). Files containing the reads have been submitted to EBI (ERA051893).

## Supporting Information

Figure S1 **Q-PCR validation of Illumina sequencing results for resting eggs (RE, top panel) and amictic eggs (AE, lower panel).** The relative abundance of twelve genes (list of primers is shown in [111]), was normalized using the equation: ratio = (E_target_)_CP_target_/(E_*ef1a*_)_CP_*reference*_ where E = 10_-1/slop_ [118]. The median was calculated for all transcript ratios and the transcript ratios were displayed as the log2[{transcript ratio of a sample}/{median of transcript ratios of all samples}]. It was very difficult to find one gene that could serve as a reference for all samples as ATP synthase (*atps*) changed between amictic eggs and resting eggs and elongation factor 1 alfa (*ef1a*) changed between females and males. Therefore, the relative abundance of transcripts was normalized to *ef1a* in the comparison made with the eggs samples but *atps* was used for comparisons between female and male samples. The expression levels obtained with real-time PCR were compared with the expression levels obtained from the number of Illumina reads for each corresponding gene. Regression analysis revealed high correlation between expression values obtained by real-time RT-PCR and number of reads for each transcript for the resting eggs (Person r = 0.980) and amictic eggs (Pearson r = 0.957). Negative values in real-time PCR indicate that the expression level of a specific transcript, was lower than that of the median. For Illumina reads, a negative value indicates that the number of reads assigned to a specific gene transcript were lower than the median value of the reads in a specific library.
(TIFF)

Table S1 **Basic information obtained by Illumina sequencing technology of libraries constructed from amictic (AE) and resting (RE) eggs.** The total number of reads was circa 16 and 14 million for the amictic and resting egg libraries, respectively. Analysis of the data was performed as described in [111] and included scaling of the data, aligning of the reads with the sequences of EST data base obtained previously [26] and filtering of ESTs with less than 50 reads per EST. Only 69% and 73% of the final ESTs showed significant matches against proteins using Blastx sequence similarity searching.
(RTF)

Table S2 **Hatching dynamics of resting eggs collected from two batches that served for RNA extraction.**
(RTF)

Table S3 **Differentially expressed transcripts in resting eggs with associated BLAST sequence similarity data and putatively assigned functions.**
(XLS)

Table S4 **Differentially expressed transcripts in amictic eggs with associated BLAST sequence similarity data and putatively assigned functions.**
(XLS)
