# Supplementary figures and images for: Long-Term Survival of Hydrated Resting Eggs from Brachionus plicatilis
Source: PLoS One. 2012 Jan 9;7(1):e29365. doi: 10.1371/journal.pone.0029365 (PMC3253786; doi:10.1371/journal.pone.0029365)

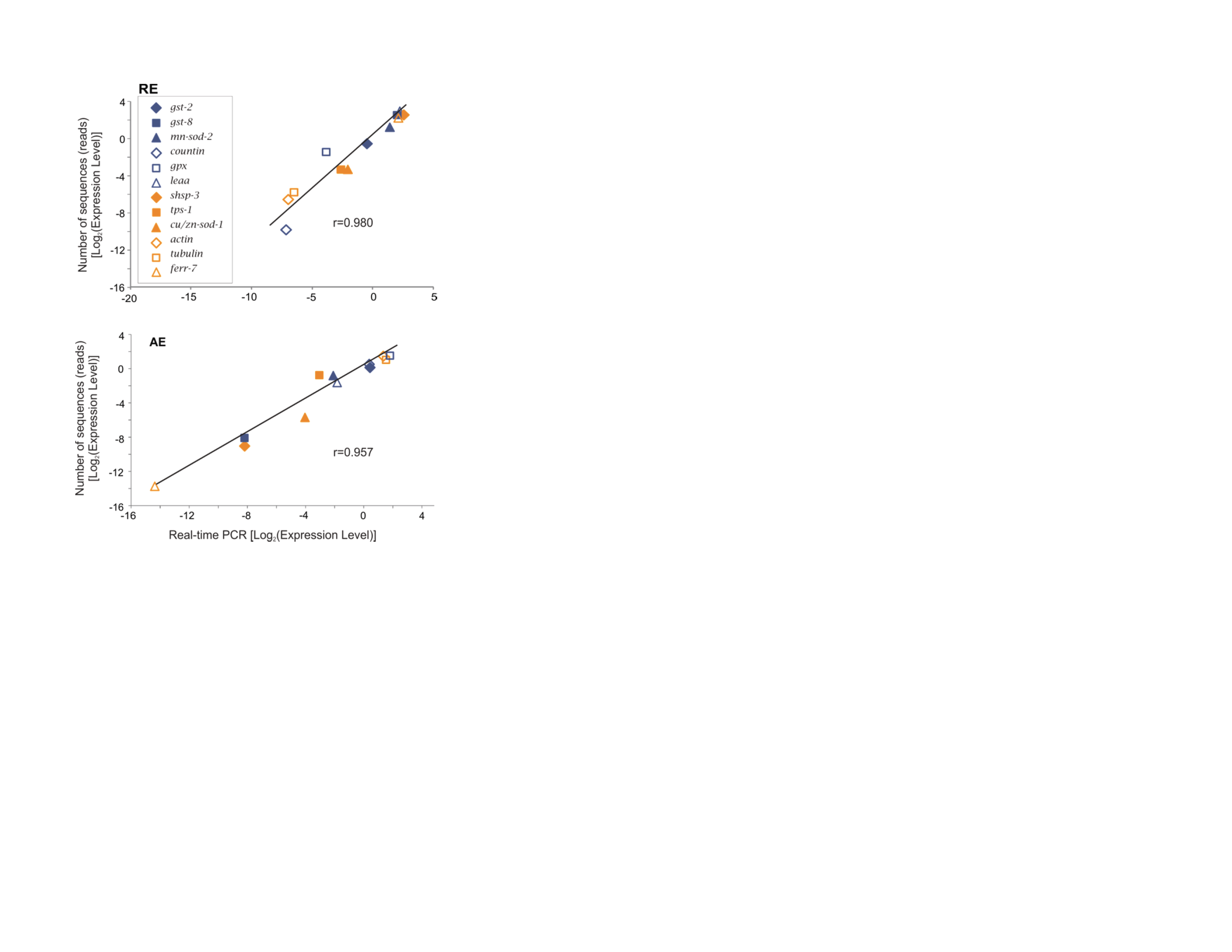

Supplement: Figure S1 — Q-PCR validation of Illumina sequencing results for resting eggs (RE, top panel) and amictic eggs (AE, lower panel). The relative abundance of twelve genes (list of primers is shown in [111]), was normalized using the equation: ratio = (Etarget)CP_target/(Eef1a)CP_reference where E = 10-1/slop [118]. The median was calculated for all transcript ratios and the transcript ratios were displayed as the log2[{transcript ratio of a sample}/{median of transcript ratios of all samples}]. It was very difficult to find one gene that could serve as a reference for all samples as ATP synthase (atps) changed between amictic eggs and resting eggs and elongation factor 1 alfa (ef1a) changed between females and males. Therefore, the relative abundance of transcripts was normalized to ef1a in the comparison made with the eggs samples but atps was used for comparisons between female and male samples. The expression levels obtained with real-time PCR were compared with the expression levels obtained from the number of Illumina reads for each corresponding gene. Regression analysis revealed high correlation between expression values obtained by real-time RT-PCR and number of reads for each transcript for the resting eggs (Person r = 0.980) and amictic eggs (Pearson r = 0.957). Negative values in real-time PCR indicate that the expression level of a specific transcript, was lower than that of the median. For Illumina reads, a negative value indicates that the number of reads assigned to a specific gene transcript were lower than the median value of the reads in a specific library. (TIFF) [file pone.0029365.s001.tiff]
