# Supplementary material for: Long-Term Survival of Hydrated Resting Eggs from Brachionus plicatilis
Source: PLoS One. 2012 Jan 9;7(1):e29365. doi: 10.1371/journal.pone.0029365 (PMC3253786; doi:10.1371/journal.pone.0029365)
Supplement: Table S1 — Basic information obtained by Illumina sequencing technology of libraries constructed from amictic (AE) and resting (RE) eggs. The total number of reads was circa 16 and 14 million for the amictic and resting egg libraries, respectively. Analysis of the data was performed as described in [111] and included scaling of the data, aligning of the reads with the sequences of EST data base obtained previously [26] and filtering of ESTs with less than 50 reads per EST. Only 69% and 73% of the final ESTs showed significant matches against proteins using Blastx sequence similarity searching. (RTF) [file pone.0029365.s002.rtf]

Supplementary Table S1: Basic information obtained by Illumina sequencing technology of libraries constructed from amictic (AE) and resting (RE) eggs.  
Description of library	AE	RE	
Total reads in the library	 16.18 x106	 14.63  x106	
Number reads in the library after scaling	9.52  x106	9.52  x106	
Reads aligned to EST backbone*	9.75 x10^6^ 	10.07  x106	
Average reads per EST	525	575	
Median of number of reads per EST	59	32	
Number of ESTs with at least 50 reads	9903	7641	
Number of ESTs with significant matches with  protein database (E-value < 1.0 E-10)	6805	5563	
% of ESTs with matches to protein data	69%	73%	


The total number of reads was circa 16 and 14 million for the amictic and resting egg libraries, respectively. Analysis of the data was performed as described in [111] and included scaling of the data, aligning of the reads with the sequences of EST data base obtained previously [26] and filtering of ESTs with less than 50 reads per EST. Only 69% and 73% of the final ESTs showed significant matches against proteins using Blastx sequence similarity searching. 
