# Supplementary material for: Long-Term Survival of Hydrated Resting Eggs from Brachionus plicatilis
Source: PLoS One. 2012 Jan 9;7(1):e29365. doi: 10.1371/journal.pone.0029365 (PMC3253786; doi:10.1371/journal.pone.0029365)
Supplement: Table S2 — Hatching dynamics of resting eggs collected from two batches that served for RNA extraction. (RTF) [file pone.0029365.s003.rtf]

Supplementary Table S2: Hatching dynamics of resting eggs collected from two batches that served for RNA extraction.
Storage period at 4°C (weeks)	Number of eggs	Number of hatched eggs 	% hatching	
0	Batch 1: 72	none	0	
	Batch 2: 72	none	0	
2	Batch 1: 73	none	0	
	Batch 2: 62	none	0	
4	Batch 1: 75	5	6.7	
	Batch 2: 74	2	2.7	
6	Batch 1: 95	29	30.5	
	Batch 2: 38	5	13.2	
8	Batch 1: 96	47	49.0	
	Batch 2: 38	18	47.4	


Resting eggs were collected 15-21 days after hatching of parental resting eggs and were distributed (an estimated  40-100 resting eggs per vial) into flat bottom cryovials containing 10ppt heat-sterilized sea water and wrapped with aluminum foil. The vials were kept in box at 4°C for 0-8 weeks. To observe hatching, the content of a vial was poured into 3.5 mm petri dish that was placed in an illuminated algal culture room at 25°C. The total number of eggs was counted and hatching was observed every after 12, 24 and 36 hr and continued every day until day 11. Moving embryos within the encased resting eggs were observed at 24 hr and the first hatched eggs were observed after 36 hrs. The hatched rotifers were removed at each observation period. Preliminary experiments showed that it takes more than 24 hr for the hatched eggs to produce amictic offspring. The cumulative number of hatched eggs and the percent of hatched eggs is shown in the table.
